# Pathways to mental well-being for graduates of mindfulness-based cognitive therapy (MBCT) and mindfulness-based stress reduction (MBSR): A mediation analysis of an RCT

**DOI:** 10.1080/10503307.2023.2269299

**Published:** 2023-11-06

**Authors:** Shannon Maloney, Jesus Montero-Marin, Willem Kuyken

**Affiliations:** 1 Department of Psychiatry, University of Oxford, Oxford, UK; 2 Research & Innovation Unit, Parc Sanitari Sant Joan de Déu, Sant Boi de Llobregat, Spain; 3 Consortium for Biomedical Research in Epidemiology & Public Health (CIBER Epidemiology and Public Health - CIBERESP), Madrid, Spain

**Keywords:** MBCT-TiF, mindfulness-based, mediation, indirect effect, mental health, well-being

## Abstract

**Objective:**

To explore mediated effects of Mindfulness-Based Cognitive Therapy-“Taking it Further” (MBCT-TiF) on mental well-being through changes in mindfulness, self-compassion, and decentering.

**Method:**

A secondary analysis of an RCT using simple mediation, with 164 graduates of MBCT and mindfulness-based stress reduction (MBSR), was implemented whereby MBCT-TiF (vs ongoing mindfulness practice; OMP) was the independent variable; changes in mindfulness, self-compassion, and decentering during the intervention were the mediators; and mental well-being at post-intervention, whilst controlling for baseline, was the dependent variable. Secondary outcomes included psychological quality of life, depression, and anxiety.

**Results:**

Compared to OMP, MBCT-TiF experienced significant improvements in mental well-being through changes in all three mediators (mindfulness: *ab *= 0.11 [0.03, 0.25]; decentering: *ab *= 0.16 [0.05, 0.33]; self-compassion: *ab *= 0.07 [0.01, 0.18]). A similar pattern was demonstrated for depression, but only mindfulness and decentering mediated effects on psychological quality of life and anxiety.

**Conclusion:**

The findings provide preliminary support for all three mediators in driving change in mental well-being in a sample of MBCT/MBSR graduates. Future work must be theory-driven and powered to test all mediators in parallel and alongside other potential mediators (e.g., equanimity) to further understand independent contributions and interacting effects.

**Trial registration:** ClinicalTrials.gov identifier: NCT05154266.

**Clinical or methodological significance of this article**: The current paper evaluates the putative mechanisms of action underlying a novel mindfulness-based cognitive therapy programme (MBCT-TiF) tailored to graduates of MBCT and mindfulness-based stress reduction (MBSR) to help enhance resilience across the mental health spectrum, from mental ill health to mental well-being. MBCT-TiF was compared to ongoing mindfulness practice (OMP). Using simple mediation models, MBCT-TiF compared OMP significantly improved mental well-being through changes in mindfulness, self-compassion, and decentering. In the context of the wider MBCT literature, this result provides further support for these processes as potential universal mechanisms that may help move the population more towards mental well-being and away from mental languishing. These findings sit within the broader global mental health context with the aim of reaching a parsimonious solution in terms of which processes drive change in mental health and well-being across the population. Formal mediation analyses that follow key testing and reporting criteria were conducted to help classify the type of mediation to help inform future work.

## Introduction

The contribution of mental ill health on total disease burden continues to grow and treatment alone cannot effectively address the prevention of mental ill health or the promotion of greater mental well-being across the population (World Health Organization, 2021). With a larger proportion of total disease burden being attributed to lower-risk cases entering ill health, without intervention, population-based approaches that aim to address the spectrum of mental health, from mental ill health to well-being, are needed (Keyes, 2002; Rose, 2008). In line with global mental health initiatives, supported by the World Health Organization (WHO) and Sustainable Development Goals (SDGs), this work is integral in the context of building protective factors and resilience on the individual, societal, global, and planetary level (Patel et al., 2018; United Nations, 2022; World Health Organization, 2022). In addition to looking for mental health approaches, that fit within this broader context, more research that aims to identify key processes or skills that drive these changes in mental health across the population is required to help reach a parsimonious solution for intervention targets (Hayes et al., 2022).

Mindfulness-based programmes (MBPs) traditionally follow eight-week formats with weekly group-based sessions, led by a trained mindfulness teacher, and include daily self-led mindfulness practice (Kabat-Zinn, 2013; Segal et al., 2018). MBPs have demonstrated promising effects in addressing mental health across a wider distribution of the population (Galante et al., 2021; Khoury et al., 2013; van Agteren et al., 2021). For example, a recent systematic and meta-analytic review evaluated a range of psychological interventions and their effects on mental well-being and found that mindfulness-based interventions demonstrated significant small to moderate improvements in general and mentally and physically ill population samples (van Agteren et al., 2021). In the context of non-clinical samples, a recent systematic review also reported small to moderate effects for MBPs, when compared to no intervention, on anxiety, depression, distress, and well-being (Galante et al., 2021). MBPs and mindfulness interventions align with global mental health initiatives because both offer upstream solutions to reduce the development of mental ill health and aim to address the spectrum of mental health and, as a result, help strengthen resilience (Oman, 2023). In light of the growing evidence base and the conceptual overlap, there is an argument for MBPs as a global mental health approach.

In terms of the theorized mechanisms of action, MBPs are believed to produce effects through the development of mindfulness skills (Feldman & Kuyken, 2019; Kabat-Zinn, 2013; Segal et al., 2018). Mindfulness, as a multidimensional construct, involves paying attention to the present-moment experience (i.e., body sensations, emotions, thoughts, and behaviours) with attitudes such as curiosity and self-compassion. It enables people to take a wider perspective (sometimes called decentering or meta-awareness) and see both internal and external stimuli has temporary events, which alongside the cultivated attitudes of mindfulness (e.g., self-compassion), allows one to respond more skilfully and with greater discernment. Past systematic reviews (Alsubaie et al., 2017; Gu et al., 2015; Maddock & Blair, 2021; Maloney et al., under review a; van der Velden et al., 2015) have provided support for these theorized mechanisms across different population samples and outcomes. Mindfulness-based cognitive therapy (MBCT) is an MBP that was originally developed to help shift the proportion of the population that is at a high risk of depressive relapse or languishing into a greater state of wellness. The theory proposes that MBCT helps reduce risk of relapse by increasing mindfulness skills and its overlapping processes (e.g., self-compassion, decentering) to help target processes (e.g., cognitive reactivity, rumination, and worry) that drive depression (Segal et al., 2018). Adapted MBCT curricula have since been developed to help reach wider audiences. These programmes retain the key elements (“the warp”), of the original MBCT for Depression protocol, but differ in terms of the more flexible components (“the weft”) to help tailor the programme to unique population samples and contexts (Crane et al., 2017). MBCT curricula adapted to more general population samples (e.g., MBCT-“Finding Peace in a Frantic World” [M-FP] (Williams & Penman, 2011) and “MBCT for Life” [MBCTL] (Strauss et al., 2021)) aim to shift a wider distribution of the population towards greater mental well-being. MBCT-“Taking it Further” [MBCT-TiF] is another adapted MBCT curriculum, which can be offered to general population samples. However, this programme differs from M-FP and MBCTL in that it is tailored to individuals who have already completed an MBP and are looking for ways to sustain improvements and extend learning. Across these adapted MBCT curricula, the idea is to provide a potential care pathway whereby individuals across the mental health spectrum (e.g., mental ill health to mental well-being) can learn foundational resilience skills to better support their mental health. This care pathway is referred to as “*the funnel*”, since the expectation is that the MBCT curricula offered to wider audiences (e.g., M-FP and MBCTL) will optimize reach but may produce smaller effects (*beginning of funnel*) whereas more tailored programmes (e.g., MBCT-TiF) will optimize effects but may reach a smaller audience (*end of funnel*) (Greenberg & Abenavoli, 2017). Past work has examined the acceptability and effectiveness of these adapted MBCT curricula in the context of improving mental health and well-being (Maloney et al., 2023; Montero-Marin et al., 2021; Strauss et al., 2021). Moreover, there is some preliminary work on the underlying mechanisms of action (Montero-Marin et al., 2021; Strauss et al., 2021). However, more replication work is required to further understand which mechanisms of action may help universally drive change across the funnel.

The parent trial paper, which was the first empirical evaluation of MBCT-TiF (Maloney et al., under review b), demonstrated that this programme was acceptable across a variety of ratings (e.g., programme expectations and credibility, teacher quality, harm and unpleasant experiences, and engagement) and was significantly more efficacious than ongoing mindfulness practice (OMP), in a sample of MBP graduates, in improving mental well-being, psychological quality of life, and symptoms of anxiety and depression. The current paper is a secondary analysis of this RCT and aims to build on these findings by investigating the mediating role of theorized processes of change (mindfulness, self-compassion, and decentering) on outcomes of mental health and well-being. Through this investigation, the hope is to further our understanding of the mechanisms that may help drive change across this funnel and effectively address the spectrum of mental health. The primary hypothesis is that the MBCT-TiF arm, when compared to the OMP arm, will experience significant improvements in mental well-being through changes in mindfulness, self-compassion, and decentering. The secondary hypotheses include the following: the MBCT arm, compared to the OMP arm, will produce significant changes in psychological quality of life and symptoms of depression and anxiety through all three theorized mediators (mindfulness, self-compassion, and decentering).

## Method

### Participants

The study population considered English-speaking adults (aged ≥18) who have already completed an MBCT/MBSR programme or any direct adaptation of these parent programmes. The exclusion criteria included: (1) current participation in a mindfulness teacher training pathway and (2) completion of MBCT-TiF.

The original sample size calculation (*n* = 164) was estimated using G*Power based on an expected medium difference between the intervention and control group on pre–post changes in mental well-being (Cohen’s *d* = 0.5), with 80% power, α = 0.05, and a dropout rate of 20%. An intention-to-treat (ITT) approach, using the Full Information Maximum Likelihood (FIML) to handle missing data, was used in the current paper for a secondary mediation analysis of the parent trial paper (Maloney et al., 2023). A post hoc power analysis based on mediation was conducted, considering the original sample size [164 cases]. This analysis estimated the product of paths “*a*” and “*b*” to approximate the power of the bootstrap estimates (Fritz & MacKinnon, 2007). With all 164 complete cases, this would allow us to identify IEs (“*ab*”) with a statistical power of 0.80, supposing the existence of intermediate effects in both paths “*a*” and “*b*” with a standardized value of 0.25 each and large effects in path “*c*” (direct effect after controlling for the indirect effects (IEs)) with a standardized value of 0.45 (Supplement A).

### Procedure

Individuals were recruited across two study cohorts in June 2021 (cohort 1) and September 2021 (cohort 2) using social media platforms (i.e., posts and newsletters), email invitations, and an existing database of MBSR/MBCT graduates. All participants who completed the eligibility form and consented were emailed the first two online surveys, automated one week apart, to establish a stable baseline [SB1-SB2]. During this period, participants were also invited to complete an online orientation video. Participants were then randomized (1:1) to MBCT-TiF or OMP and were asked to complete an online survey right at the start of the twelve-week study period (week 0; T0), twice during (week 4 and 8; T1-T2), and once immediately after the end of the study period (week 12; T3). The online surveys were managed remotely and automated using *Qualtrics*. For the CONSORT flow diagram and more details regarding the procedure, see parent trial paper (Maloney et al., under review b).

A member of the study management team, not involved in data collection or analysis, created the randomization list and allocation was concealed from the experimenter during the study period. The study was reviewed and approved by the Medical Science Division Research Ethics Committee at the University of Oxford (R75514/RE001; 12/05/2021). All participants provided written informed consent prior to the start of the study. As a form of compensation for taking part, the MBCT-TiF course was significantly subsidized and offered at a 50% discounted rate. The parent trial was registered with ClinicalTrials.Gov (NCT05154266; 13/12/2021). As a secondary analysis of this parent trial, the mediation analyses are presented as exploratory.

MBCT-TiF was offered online as a twelve-week programme with weekly group-based sessions for around 135 min each, led by a trained mindfulness teacher, and included daily self-led mindfulness practice for around 30-45 min per day. The programme was offered by certified mindfulness teachers who taught at least one MBCT-TiF programme and met good practice criteria (BAMBA; https://bamba.org.uk/). The OMP group was offered the MBCT-TiF programme at the end of the study period but no additional data was collected. The majority (around 74%) of the OMP group, during the twelve-week study period, continued their self-led mindfulness practice for around 15 min per day (Maloney et al., under review b).

### Instruments

Participants were invited to complete online questionnaires to evaluate sociodemographic characteristics; psychological outcomes [mental well-being, symptoms of depression and anxiety, psychological quality of life]; acceptability outcomes [expectations, credibility, teacher quality, potential unpleasant experiences and harm, and engagement (total attendance and amount of self-led mindfulness practice)]; and potential mechanisms [mindfulness, decentering, self-compassion]. The original trial paper (Maloney et al., under review b) provides the results for the psychological outcomes and acceptability outcomes pre–post-intervention. The current paper will address potential mechanisms assessed during the twelve-week study period [weeks 4 and 8; T1-T2] in relation to the primary outcome (mental well-being) and secondary outcomes (psychological quality of life and symptoms of depression and anxiety) at week 12 (post-intervention; T3) whilst controlling for baseline levels (T0).

The primary mechanism (mindfulness) was assessed using the 15-item Five-Facet Mindfulness Questionnaire-Short Form (FFMQ-SF; Baer et al., 2006; Gu et al., 2016). The total score was calculated, without the observing subscale, which has been recommended by Gu et al. (2016) when considering changes of MBCT pre–post intervention. Items were responded to on a scale of 1 (*never or very rarely true*) to 5 (*very often or always true*). Cronbach’s alpha values were: T0: 0.90; T1: 0.89; T2: 0.90; and T3: 0.91. The secondary mechanisms were constructs relating to decentering and self-compassion. Decentering was measured using the 11-item Experience Questionnaire (EQ; Fresco et al., 2007). The total mean score was calculated and items were responded to on a scale of 1 (*never*) to 5 (*all the time*). Cronbach’s alpha values were: T0: 0.90; T1: 0.91; T2: 0.93; and T3: 0.94. Self-compassion was measured using the 12-item Self-Compassion Scale Short Form (SCS-SF; Raes et al., 2011). The total mean score was calculated and items were responded to on a scale of 1 (*almost never*) to 5 (*almost always*). Cronbach’s alpha values were: T0: 0.91; T1: 0.91; T2: 0.93; and T3: 0.93.

The primary outcome (mental well-being) was evaluated using the 14-item Warwick Edinburgh Mental Well-being (WEMWBS; Tennant et al., 2007) questionnaire. The total score was calculated and items were responded to on a scale of 1 (*none of the time*) to 5 (a*ll of the time*). Cronbach’s alpha values were: T0: 0.93; T1: 0.94; T2: 0.94; and T3: 0.95. The secondary outcomes included psychological quality of life and symptoms of anxiety and depression. Psychological quality of life was assessed using the 6-item psychological domain of the World Health Organization Quality of Life (BREF) measure (WHOQOL-BREF; World Health Organization, 1996) with the total transformed score (0-100) calculated and items responded to on a scale of 1 (*not at all*) to 5 (*an extreme amount*). Cronbach’s alpha values were: T0: 0.83; T1: 0.81; T2: 0.85; and T3: 0.87. Symptoms of depression were assessed using the 9-item Patient Health Questionnaire (PHQ-9; Kroenke et al., 2001). The total score was calculated and items were responded to on a scale of 0 (*not at all*) to 3 (*nearly every day*). Cronbach’s alpha values were: T0: 0.83; T1: 0.84; T2: 0.87; and T3: 0.85. Symptoms of anxiety were evaluated using the 7-item Generalized Anxiety Disorder (GAD-7; Spitzer et al., 2006) questionnaire. The total score was calculated and items were responded to on a scale of 0 (*not at all*) to 3 (*nearly every day*). Cronbach’s alpha values were: T0: 0.87; T1: 0.91; T2: 0.89; and T3: 0.91.

### Statistical Analysis

Means (standard deviations) or frequencies (percentages) were used to describe the sociodemographic and psychological characteristics in the total group and across conditions at baseline (T0). The corresponding *t*-test for continuous variables or *χ*^2^ for categorical variables were used for comparisons between conditions. Bivariate Pearson’s correlations were calculated at T0 to determine potential overlap among constructs at baseline and at T1-T2 to understand the extent to which change in one mediator was accompanied by change in another mediator during the intervention. Partial correlations between the amount of self-led mindfulness practice and the primary outcome (mental well-being) and mediator (mindfulness) at T3, after controlling for baseline levels [T0], were also calculated within both the MBCT-TiF and OMP group to address the gradient criterion to help establish a mechanism of change (Kazdin, 2007; Maloney et al., under review a).

Mediation path analytic models were performed to test the primary hypothesis, whereby mediators constitute the interim processes between the independent variable (IV) and dependent variable (DV) and are established statistically. Mechanisms, on the other hand, are conceptualized as the same but are only established over time and once key requirements have been met (Kazdin, 2007). For the testing and reporting requirements for mediation, the current paper has prioritized the Zhao et al. (2010) framework. This framework prioritizes the IE to test mediation and puts forward testing and reporting requirements which help categorize the type of mediation [complementary, competitive, indirect-only, direct-effects, and no effect] to help inform future work. According to this framework, the IE constitutes mediation and refers to the effect of the IV on the DV through the mediator variable. It measures the extent to which the IV influences the DV indirectly through the mediating variable, rather than through a direct pathway [the direct effect]. Conversely, the direct effect (DE) represents this direct pathway between the IV and DV, after controlling for the mediator. For a visual depiction of the IE and DE, see Supplement B. For a full description of this framework and the different mediation classifications, see Zhao et al. (2010).

In the current paper, the mediation analysis implemented a univariate model whereby each proposed mediators is tested in isolation and independent of the others. The justification for using univariate mediation models, rather than parallel (multivariate) mediation models, concerns issues around statistical power, with a larger sample size required in light of the number of parameters to be estimated (Xu et al., 2020) [see Supplement A]. The group condition (i.e., “MBCT-TiF” vs. “OMP”) was considered as the independent variable. Potential mediators (i.e., mindfulness, decentering, and self-compassion) were computed as change over time during the study period (T1-T2). For that, we estimated standardized residualised change scores using a simple linear regression model in which T1 (week 4) scores predicted T2 (week 8) scores. The standardized residuals were then used in the mediation analyses. We examined the standardized primary (i.e., mental well-being) and secondary (i.e., psychological quality of life, anxiety, depression) outcomes at T3 (week 12) and covaried the outcome at baseline (T0). Potential covariates (age, gender, cohort, and previous type of mindfulness course) were considered, but were not included in light of no significant differences found at baseline across groups (MBCT-TiF vs OMP). The primary mediation analyses used maximum likelihood regression path analysis and prioritized an ITT approach using FIML with the Expected Maximization Algorithm (EMA) and Montecarlo integration to address missing data. To explore potential reverse mediation, we carried out sensitivity analyses using the same approach. Regression coefficients of bootstrapped IEs (*ab*) were estimated, as well as their 95% confidence intervals (95% CIs), based on 10,000 bootstrap samples. A significant mediating effect was considered when the bootstrapped 95% CI did not include zero (Lockhart et al., 2011). The percentage of variance in the outcome that was explained by the mediating model was established by means of determination coefficients (*R*^2^). An alpha level of 0.05 was set, using a two-tailed test. Given the exploratory nature of this study, we did not correct for multiple testing. The statistical packages used were SPSS v29.0 and R v4.2.2.

In addition to following the reporting and testing requirements, established by Zhao et al. (2010), the current paper also considers additional criteria that do not conflict with the Zhao et al. (2010) framework that were originally proposed by Kazdin (2007) and explored further in the context of mindfulness research by Alsubaie et al. (2017) and Maloney et al. (under review a). Additional criteria that were considered in the current paper include the “*plausibility or coherence*” criterion, which looks at the extent to which the broader literature supports the likelihood of the proposed mechanisms; the “*timeline*” criterion, which argues that change in the proposed mechanism should occur before change in outcome; and the “gradient” criterion, which in the context of mindfulness research (Maloney et al., under review a) considers the (a) association between the amount of practice completed and change in the proposed mediator and (b) an association between the amount of practice completed and change in outcome. Given that the amount of mindfulness practice (dosage) is arguably one medium through which change occurs (Parsons et al., 2017), the extent to which these associations are established can help increase confidence in the extent to which the putative mechanism is mindfulness-specific.

## Results

The sociodemographic and psychological characteristics of participants at baseline can be seen in Table I. Most participants were female (69.5%), in their early fifties (M(SD) = 50.55 (12.70)), from the UK (67.7%), employed (74.4%), with an average of 3.23 (SD = 3.46) years since completing their previous mindfulness course. Characteristics were similar between groups at baseline, with no significant differences in relation to sociodemographic and psychological variables.

**Table I.** Baseline characteristics of participants

	MBCT-TiF (*n* = 83)	OMP (*n* = 81)	Total (*n* = 164)
Age, mean (SD)	50.52 (12.50)	50.59 (12.98)	50.55 (12.70)
Gender			
Female, n (%)	56 (67.5)	58 (71.6)	114 (69.5)
Male, n (%)	26 (31.3)	23 (28.4)	49 (29.9)
Country			
UK, *n* (%)	56 (67.5)	55 (67.9)	111 (67.7)
Others, *n* (%)	27 (32.5)	26 (32.1)	53 (32.3)
Occupation			
Employed, *n* (%)	59 (71.1)	63 (77.8)	122 (74.4)
Unemployed, *n* (%)	4 (4.8)	2 (2.5)	6 (3.7)
Student, *n* (%)	6 (7.2)	5 (6.2)	11 (6.7)
Retired, *n* (%)	11 (13.3)	10 (12.3)	21 (12.8)
Previous mindfulness course			
MBCT, *n* (%)	60 (72.3)	60 (74.1)	120 (73.2)
MBSR, *n* (%)	23 (27.7)	21 (25.9)	44 (26.8)
Group delivery			
Cohort 1, *n* (%)	41 (49.4)	40 (49.4)	81 (49.4)
Cohort 2, *n* (%)	42 (50.6)	41 (50.6)	83 (50.6)
Years since course, mean (SD)	3.65 (3.45)	2.76 (3.44)	3.23 (3.46)
Mental well-being, mean (SD)	46.58 (9.97)	46.86 (7.40)	46.72 (8.77)

Note. Table includes the baseline characteristics of the ITT sample across groups using means (SD) or frequencies (%), depending on the distribution of the variable. For gender, there was one case in the MBCT-TIF group and no cases in the CONTROL group that identified as “other”. For occupation, there were three cases in the MBCT-TIF and one case in the CONTROL group that was missing. For previous mindfulness course (total sample), 44.4% reported the original MBCT for Depression protocol, 25.9% reported MBSR, 23.5% reported MBCTL, 3.7% reported M-FP, and 2.5% reported “other” type of MBCT protocol. A sub-group of the participants completed the question regarding years since completing an MBCT/MBSR course (*n* = 98). The baseline mental well-being scores with the ITT sample was taken at SB1 time point. MBCT-TiF: Mindfulness-Based Cognitive Therapy – Taking it Further; OMP: Ongoing Mindfulness Practice.

The correlations between the potential mediators and outcomes at baseline [T0] (Supplement C) revealed a large convergence between outcomes (Pearson’s r range in absolute value from 0.57–0.78), as well as between potential mediators (Pearson’s r range from 0.72–0.76), and between outcomes and potential mediators (Pearson’s r range in absolute value from 0.39–0.69). The correlations between week 4 and 8 [T1-T2] residualised change scores in the potential mediators were large (“mindfulness-decentering” r = 0.52, *p* < 0.001; “mindfulness-self-compassion” r = 0.51, *p* < 0.001; “decentering-self-compassion” r = 0.66, *p* < 0.001), suggesting that improvements in each potential mediator during the programme were accompanied by improvements in the other potential mediators. In the MBCT-TiF arm, the correlations between total amount of self-led mindfulness practice between week 4 and 12 [T1-T3] and mental well-being at T3, after controlling for baseline levels at T0, were moderate (r = 0.38, *p* = 0.002). The correlations between total amount of self-led mindfulness practice [T2-T4] and mindfulness [T4], after controlling for baseline levels [T1], were also moderate (r = 0.36, *p* = 0.004). In the OMP arm, the correlations between the total amount of self-led mindfulness practice between week 4 and 12 [T1-T3] and mental well-being at T3, after controlling for baseline levels at T0, were absent (r = 0.04, *p* = 0.764). The correlations between total amount of self-led mindfulness practice [T2-T4] and mindfulness [T4], after controlling for baseline levels [T1], were also absent (r = −0.01, *p* = 0.913).

The results of the path analyses on the primary outcome (mental well-being) are detailed in Table II. The three models that were tested (i.e., mindfulness, decentering, and self-compassion as mediators of the effect of the intervention on the outcome) showed a significant indirect effect (mindfulness: *ab *= 0.11 (bootstrapped 95% CI = 0.03, 0.25), decentering: *ab *= 0.16 (bootstrapped 95% CI = 0.05, 0.33), self-compassion: *ab *= 0.07 (bootstrapped 95% CI = 0.01, 0.18))). The direct effect (*path c*) of the intervention, after controlling for the mediators, was significant and of the same sign as the IEs in the three models.

**Table II.** Direct and bootstrapped indirect effects in the simple mediation path analysis models on mental well-being

Mediators	*R^2^*	*p^a^*	Direct effects			Indirect effects		
			*path*	*Coeff.*	*p^b^*	*path*	*Coeff.*	*95%CI*
Mindfulness	0.22	0.001	*a*	0.49	0.002	*ab*	0.11	0.03, 0.25
			*b*	0.23	0.002			
			*c*	0.71	<0.001			
			*c’*	0.82	<0.001			
Decentering	0.23	<0.001	*a*	0.59	<0.001	*ab*	0.16	0.05, 0.33
			*b*	0.26	0.002			
			*c*	0.66	<0.001			
			*c”*	0.81	<0.001			
Self-compassion	0.19	0.002	*a*	0.44	0.007	*ab*	0.07	0.01, 0.18
			*b*	0.15	0.029			
			*c*	0.76	<0.001			
			*c’*	0.63	<0.001			

An intention-to-treat (ITT) approach was used, using the Full Information Maximum Likelihood (FIML) to address missing data. The independent variable is the group condition (MBCT-TiF vs OMP). The potential mediator (mindfulness, decentering, or self-compassion) was based on T1-T2 [week 4 to week 8] residualised change scores. The dependent variable (outcome) is mental well-being of life at T3 [week 12]. Models controlled for the outcome at baseline [T0]. Path coefficients are (standardized) ordinary least squares-based regression coefficients. *a*: direct path between the independent variable and the mediator. *b*: direct path between the mediator and the outcome. The product of “*ab*” is the bootstrapped indirect effect (IE) using 10,000 samples. *c*: direct effect of the independent variable on the dependent variable after adjustment for the mediating effects. *c*’: total effects. *R*^2^: variance explained by regression models. *F*: Snedecor’s *F* associated with the adjustment of the regression model. *Coeff*: (standardized) slope. *t*: Student’s *t* associated with the slope using the Wald test. *SE*: standard error. *p*^a^: *p*-value related to *F*-test. *p*^b^: *p*-value related to *t*-test. 95% CI: 95% confidence interval.

The results of the path analyses on the secondary outcomes are detailed in Supplements D, E, and F. The three models showed a significant indirect effect on depression (mindfulness: *ab *= −0.11 (bootstrapped 95% CI = −0.26, −0.03); decentering: *ab *= −0.11 (bootstrapped 95% CI = −0.27, −0.01); self-compassion: *ab *= −0.10 (bootstrapped 95% CI = −0.25, −0.02))). However, only mindfulness and decentering presented a significant indirect effect on psychological quality of life (mindfulness: *ab *= 0.13 (bootstrapped 95% CI = 0.04, 0.27); decentering: *ab *= 0.16 (bootstrapped 95% CI = 0.05, 0.32); self-compassion: *ab *= 0.08 (bootstrapped 95% CI = −0.001, 0.22))) and on anxiety (mindfulness: *ab *= −0.09 (bootstrapped 95% CI = −0.23, −0.01); decentering: *ab *= −0.14 (bootstrapped 95% CI = −0.32, −0.03); self-compassion: *ab *= −0.05 (bootstrapped 95% CI = −0.17, −0.01))). The direct effect of the intervention on the secondary outcomes [psychological quality of life, depression, and anxiety] after controlling for the mediators was significant and of the same sign to the IEs in the three models. For the sensitivity analyses that summarize the reverse mediation see Supplements G, H, and I.

## Discussion

The current paper is a mediation analysis of an RCT that compared MBCT-TiF to OMP in a sample of MBCT/MBSR graduates. The primary aim was to evaluate the mediating role of mindfulness, self-compassion, and decentering in improving mental well-being. The findings of the present paper support the primary hypothesis that MBCT-TiF, when compared to OMP, promotes change in mental well-being through improvements in all theoretically proposed mechanisms (mindfulness, self-compassion, and decentering). A scoping review (Maloney et al., under review a) identified consistent evidence for mindfulness skills and preliminary evidence for its overlapping processes (self-compassion and decentering) as potential mechanisms of action underlying MBPs in the context of mental ill health and languishing outcomes (e.g., mental health disorder, anxiety and depression symptoms, stress, and burnout), which builds off past systematic reviews and meta-analyses (Alsubaie et al., 2017; Gu et al., 2015; Maddock & Blair, 2021; van der Velden et al., 2015). However, very few studies met the testing and reporting requirements for evaluating mechanisms in the context of well-being outcomes. Therefore, the results of the current paper build on this body of work and suggest that these mechanisms may also help shift the population in a more positive direction (i.e., improved mental well-being and quality of life). In the context of the wider MBCT literature, the results provide preliminary support for these mechanisms as universal targets for addressing the spectrum of mental health, from mental ill health to well-being.

The results of this paper partially supported the secondary hypotheses that MBCT-TiF would produce change in symptoms of depression and anxiety and psychological quality of life through all proposed mechanisms of change. MBCT-TiF compared to OMP had an IE on depression through all three processes and, therefore, this hypothesis was supported. However, MBCT-TiF compared to OMP only had an IE on anxiety and psychological quality of life through mindfulness and decentering. In light of this paper being a secondary analysis of the parent paper trial (Maloney et al., under review b), whereby the original power calculation was based on the primary research question of investigating efficacy of MBCT-TiF compared to OMP, the non-significant findings for self-compassion in relation to psychological quality of life and symptoms of anxiety could be a result of low statistical power (Supplement A) and future work will need to explore this theorized mechanism in a larger sample size (Xu et al., 2020). A scoping review (Maloney et al., under review a) provided preliminary support for the mediating role of self-compassion across the mental health spectrum (mental ill health to well-being). However, none of the included papers evaluated mechanisms in the context of an adapted mindfulness-based programme, such as MBCT-TiF, for graduates of MBCT/MBSR. Therefore, future work will need to replicate these findings to further understand the role of self-compassion across the MBCT funnel and in relation to different mental health outcomes.

The mediation results specifically indicated that MBCT-TiF had a positive direct effect on change in mental well-being as well as a positive IE through the three proposed mechanisms. According to the framework established by Zhao et al. (2010), a significant IE and direct effect of the same sign (positive or negative) is called “complementary mediation,” which suggests theoretically that an additional mediator, not tested in the current model, could help explain effects. Given that each proposed mechanism was tested separately, using simple (univariate) mediation models, it may be that all three proposed mechanisms together would best explain the effects of MBCT-TiF if tested in a parallel (multivariate) mediation model. An alternative explanation could be that an additional mediator, not explored in the current paper, could help explain the effectiveness of MBCT-TiF. In a recent trial (Montero-Marin et al., 2021), that compared an instructor-led to a self-led format of the M-FP programme (Williams & Penman, 2011), significant IEs on mental well-being through mindfulness and self-compassion were reported in the context of a simple mediation model. With non-significant direct effects found, this “indirect-only” mediation suggests that it is unlikely that an additional mediator is missing from the mediation model. Therefore, these findings suggest that the model of change may require that these theorized mechanisms operate in parallel or in addition to other proposed mechanisms to produce change in the context of MBCT-TiF. Past reviews (Alsubaie et al., 2017; Gu et al., 2015; Maddock & Blair, 2021; Maloney et al., under review a; van der Velden et al., 2015) on the mechanisms underlying mindfulness-based programmes have reflected the limited evidence on how these processes interact and operate in parallel to produce change in mental health and well-being. Therefore, future work that implements parallel (multivariate) mediation models is needed.

Taking into account the strong inter-correlations between these theorized processes, the mediation findings may also be limited to the measurements ability to detect more nuanced differences across these constructs. If the current measurements are not sensitive enough this then limits our interpretation of key processes of change. An alternative explanation for why there are strong inter-correlations between the theorized mechanisms could be a result of what a recent review (Fancourt et al., 2021) refers to as inherent positive feedback loops. Fancourt et al. (2021) suggest that mechanisms in the context of complex interventions are non-linear and can include positive feedback loops whereby different mechanisms can reinforce each other which would lead to outputs that function as inputs. In regards to the proposed theory of MBCT, which suggests that there are multiple processes at play that enact change, the possibility of there being positive feedback loops between change in one mechanism and another and also between change in mechanisms and outcomes seems plausible. This model of more dynamic change can be considered with statistical approaches such as cross-lagged panel mediation, which can be considered in future research. In an effort to further explore temporality, we did include reverse mediation (Supplements G-I) which suggest that changes in mental well-being and psychological quality of life mediate change in mindfulness, self-compassion, and decentering. However, future work will need to explore reverse mediation whereby the primary aim is to explore mediation. To help unpick the extent to which the overlap is a result of conceptual blurriness or inherent positive feedback loops, future work can consider investigating several measurements that aim to measure similar constructs and more complex statistical approaches (e.g., cross-lagged panel mediation models). However, with more parameters, the required sample size increases and more exploratory work like the current paper can help pin-point which variables may be of particular interest.

In MBCT-TiF, learning how to befriend and bring a sense of appreciation and joy towards life is thought to be a specific aim of the programme. This study addresses key putative mechanisms of MBCT (e.g., mindfulness, self-compassion, decentering) and provides support for their mediating role in relation to mental well-being. However, since MBCT-TiF builds on former MBPs in regards to the dimension of cultivating attitudes of mindfulness (e.g., befriending, equanimity, appreciation, joy, kindness), future work should also consider the mediating role of these variables in the context of mental health and well-being outcomes. Moreover, as pre-mentioned, these alternative mediators should be examined in isolation and in combination with other theoretically proposed mediators (i.e., mindfulness, self-compassion, and decentering) to help further understand independent contributions and interacting effects. Future work can also consider investigating the sequence of change across the programme, with more time-points during the intervention [i.e., on weekly basis], to further investigate issues regarding temporality.

In terms of limitations, the current paper is a secondary mediation analysis of an RCT (Maloney et al., under review b), which means that the original power calculation was driven by the original research questions and represents an exploratory evaluation of mediation which can help drive future work in this area. In light of this limitation and the reported findings for complementary mediation, the current paper could not explore the interaction effects of the three proposed mediators in a parallel mediation model. Additionally, given that the paper prioritized mechanisms that are theorized to be MBCT-specific, an exploration of common factors (e.g., psychoeducation, teacher inquiry, and group effect) were not explored. Mental health approaches share some of these common factors and more research is needed in this area to further understand the independent contribution in producing change in outcome (Goldberg, 2022). Another limitation was that the sample was predominately female and from the United Kingdom. Past research has acknowledged the issue of homogeneity in psychology research (Henrich et al., 2010) and more efforts to get a more diverse sample (e.g., race, ethnicity, gender, age, etc.) involved in research is incredibly important in terms of advancing our understanding of the extent to which MBPs may serve as a potential global mental health approach. Lastly, in light of this sample being graduates of MBCT/MBSR and likely more familiar with the language used to describe mindfulness, the effects could be partially explained by this increased familiarity with the terminology. Although, changes in the theorized mechanisms were unique to the MBCT-TiF arm, versus OMP, whereby both groups consisted of graduates and presumably were both familiar with the constructs, future work should also consider integrating more objective measures in addition to self-report to help rule out this potential bias.

Strengths of the current paper include meeting the testing and reporting requirements for formal mediation methods, following the Zhao et al. (2010) framework, and exploring additional requirements for establishing a mechanism of change that do not conflict with the Zhao et al. (2010) framework (Maloney et al., under review a). These additional criteria include plausibility and coherence; timeline; and gradient. The plausibility and coherence criterion was met in the current paper by exploring mediators that are supported by the proposed theoretical framework of MBCT. The timeline criterion was established utilizing different time points for the mediators and outcomes without any overlap to address temporal precedence (Kazdin, 2007). However, this criterion may also need some refinement if future work is able to demonstrate the likelihood of positive feedback loops (i.e., dynamic change). The gradient criteria, which essentially looks at a dose–response relationship, can also be reformulated as a way to address mechanism specificity in this context. For instance, with the current paper demonstrating a relationship between change in the amount of self-led practice (dose) and the proposed mediator and outcome, and weaker associations within the OMP group, this helps increase confidence in these processes of change being specific to MBCT-TiF rather than OMP. Other criteria for establishing a mechanism of change have been put forward (Kazdin, 2007). However, some of the criteria (e.g., strong association and specificity) do conflict with the Zhao et al. (2010) testing and reporting requirements or the theory of MBCT and therefore need to be refined. In a recent scoping review (Maloney et al., under review a), the *strong association* and *specificity* criterion, as they are originally articulated, are said to conflict with the Zhao et al. (2010) framework, with the former relying on the Baron and Kenny method which does not prioritize the IE and the latter suggesting that increased confidence in a mechanism of action is contingent on ruling out other potential mechanisms which may be problematic in the context of MBCT in light of the proposed theory suggesting overlapping processes and the possibility of inherent positive feedback loops. A refinement of this criteria could involve amending the language; for instance, the specificity criterion can be about specifying mechanisms that may be unique to MBCT or a specific outcome. Data analytic approaches, like the one implemented in the current study, which appreciate the grouping variable (MBCT-TiF vs OMP) can help increase one’s understanding of mechanism specificity in this way. Ultimately, this is an area of work that requires collaborative effort and replication to further understand the utility of the proposed criteria in the context of establishing a mechanism of action in the context of MBCT.

Overall, in the context of the wider MBCT literature, the findings of the current paper provide support for mindfulness, self-compassion, and decentering as potential universal mechanisms that drive change in mental well-being across the MBCT funnel. The results also suggest that the model of change may require that these mechanisms operate in parallel or that additional mechanisms not explored in the current study may also be important in the context of MBCT-TiF. This work builds on this larger body of work that suggests that MBCT could be an accessible and effective approach that targets key mechanisms of action that drive change across a wider distribution of the population. Ultimately, global mental health initiatives need to identify key processes of change that drive change in mental well-being and MBCT has demonstrated potential as one key offering. This area of work that aims to uncover mechanisms of action is incredibly vital to increasing understanding of intervention targets that can be targeted and scaled-up to help reach wider audiences whilst at the same time optimizing effectiveness. It is likely that different global mental health approaches share similar (universal) mechanisms and, therefore, reaching a parsimonious solution can help prioritize the skills required to enact change and can help the wider field of psychotherapy research move away from prioritizing one approach over another.

## Supplementary Material

tpsr-2023-0096-File001
