# Supplementary material for: Pathways to mental well-being for graduates of mindfulness-based cognitive therapy (MBCT) and mindfulness-based stress reduction (MBSR): A mediation analysis of an RCT
Source: Psychother Res. 2023 Nov 6;34(8):1162–73. doi: 10.1080/10503307.2023.2269299 (PMC11537299; doi:10.1080/10503307.2023.2269299)
Supplement: tpsr-2023-0096-File001 [file TPSR_A_2269299_SM6302.docx]

**Pathways to mental well-being for graduates of MBCT/MBSR: A mediation analysis of an RCT**

*Supplementary Information*

**Contents**

**Supplement A:** Power analysis for causal mediation analysis……………………………………...…2

**Supplement B:** Visual depiction of the indirect effect (IE) and direct effect (DE)……………..……..3

**Supplement C**: Pearson’s correlations between the outcomes and potential mediators at T0…………………………………………………………………………………………………….....4

**Supplement D**: Direct and bootstrapped indirect effects in the simple mediation path analysis models of psychological quality of life………………………………….…………………………………........5

**Supplement E**: Direct and bootstrapped indirect effects in the simple mediation path analysis models of depression…………………………………………………….………………..............................….6

**Supplement F:** Direct and bootstrapped indirect effects in the simple mediation path analysis models of anxiety ………………….……………………………………………………………………...…….7

**Supplement G.** Direct and bootstrapped indirect effects in the simple (reversed) mediation path analysis models on mindfulness………………………………………………………………….……..8

**Supplement H.** Direct and bootstrapped indirect effects in the simple (reversed) mediation path analysis models on decentering………………………………………………………………..……..…9

**Supplement I.** Direct and bootstrapped indirect effects in the simple (reversed) mediation path analysis models on self-compassion…………………………………………………………………………….10

**Supplement A**. Power Analysis for Causal Mediation Analysis

|  |  |
| --- | --- |
| 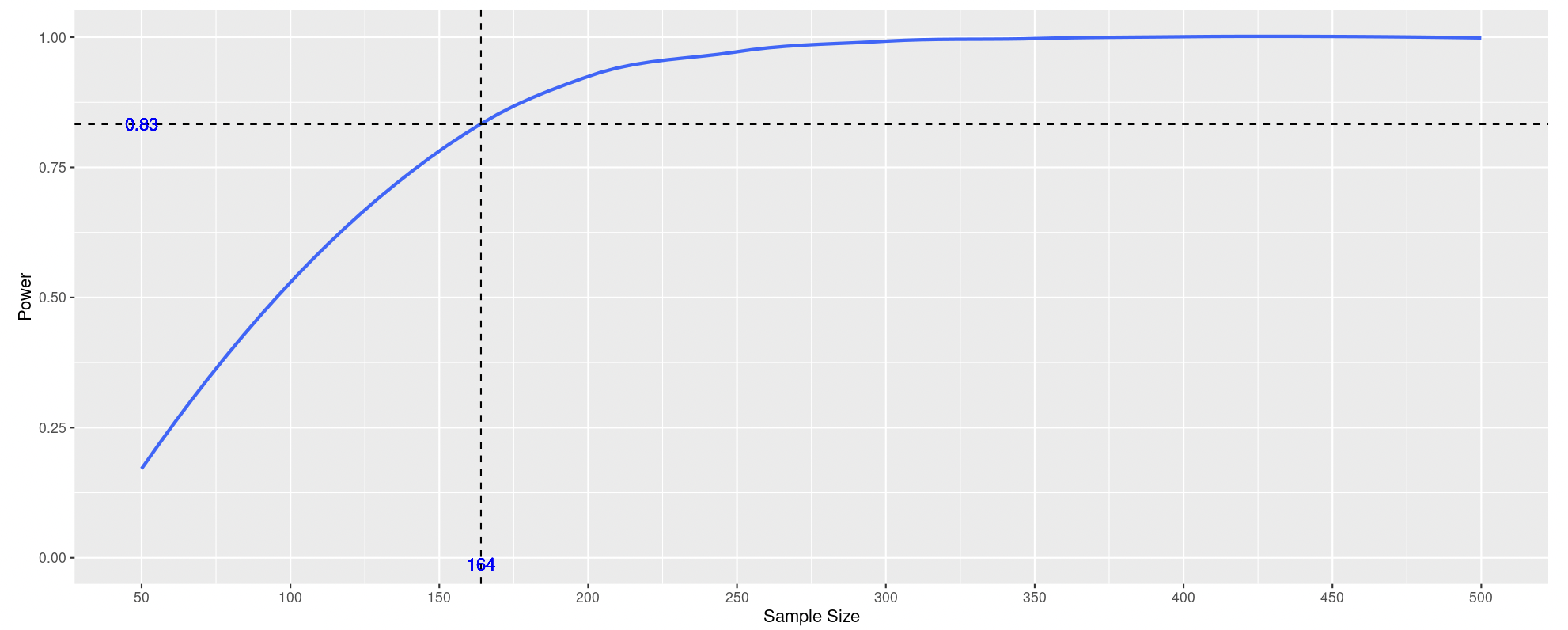 | 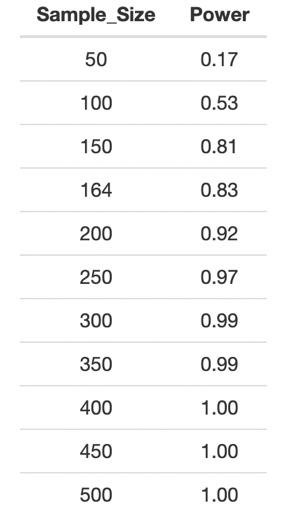 |

164 participants allow us to identify indirect effects (“*ab*”) with a statistical power of around 0.80, and a significance level of 0.05, supposing the existence of intermediate effects in both paths “*a*” and “*b*” with a standardised value of 0.25 each, and moderately large effects in path “*c*” (direct effect after controlling for the indirect effects) with a standardised value of 0.45.

**Supplement B.** Visual depiction of the indirect effect (IE) and direct effect (DE)

*Path ab* = Indirect effect

*Path c* = Direct effect

*Path c*’ = Total effect

Mediator (M)

*Path b*

*Path a*

Group (MBCT-TiF vs OMP) [IV]

Outcome (DV)

This figure aims to depict how to test the indirect effect (the product of path a and b; path ab). Path c is the direct effect between the IV (MBCT-TiF vs OMP) and the DV (outcome). Path c’ is the total effect and represents the product of paths a, b, and c. Note. IV = independent variable; DV = dependent variable; MBCT-TiF = Mindfulness-Based Cognitive Therapy-“Taking it Further’; OMP = Ongoing Mindfulness Practice; M = mediator.

*Path c*

**Supplement C**. Pearson’s correlations between the outcomes and potential mediators at T0

|  | **1** | **2** | **3** | **4** | **5** | **6** | **7** |
| --- | --- | --- | --- | --- | --- | --- | --- |
| 1. Well-being |  |  |  |  |  |  |  |
| 2. Quality of Life | 0.78*** |  |  |  |  |  |  |
| 3. Depression | -0.68*** | -0.63*** |  |  |  |  |  |
| 4. Anxiety | -0.57*** | -0.59*** | 0.71*** |  |  |  |  |
| 5. Mindfulness | 0.69*** | 0.59*** | -0.55*** | -0.52*** |  |  |  |
| 6. Decentering | 0.61*** | 0.54*** | -0.39*** | -0.44*** | 0.76*** |  |  |
| 7. Self-compassion | 0.64*** | 0.65*** | -0.49*** | -0.59*** | 0.72*** | 0.75*** |  |

***p<0.001

**Supplement D**. Direct and bootstrapped indirect effects in the simple mediation path analysis models of psychological quality of life

|  |  |  |  | | **DIRECT EFFECTS** | | | |  | **INDIRECT EFFECTS** | | | |
| --- | --- | --- | --- | --- | --- | --- | --- | --- | --- | --- | --- | --- | --- |
| **Mediators** | ***R^2^*** | ***p^a^*** | | ***path*** | | ***Coeff.*** | ***p^b^*** |  | | | ***path*** | ***Coeff.*** | ***95%CI*** |
| Mindfulness | 0.25 | 0.001 | | *a* | | 0.50 | 0.001 |  | | | *ab* | 0.13 | 0.04, 0.27 |
|  |  |  | | *b* | | 0.26 | 0.002 |  | | |  |  |  |
|  |  |  | | *c* | | 0.72 | <0.001 |  | | |  |  |  |
|  |  |  | | *c’* | | 0.85 | <0.001 |  | | |  |  |  |
| Decentering | 0.24 | <0.001 | | *a* | | 0.61 | <0.001 |  | | | *ab* | 0.16 | 0.05, 0.32 |
|  |  |  | | *b* | | 0.26 | 0.006 |  | | |  |  |  |
|  |  |  | | *c* | | 0.68 | <0.001 |  | | |  |  |  |
|  |  |  | | *c’* | | 0.84 | <0.001 |  | | |  |  |  |
| Self-compassion | 0.22 | 0.001 | | *a* | | 0.45 | 0.005 |  | | | *ab* | 0.08 | -0.001, 0.22 |
|  |  |  | | *b* | | 0.18 | 0.080 |  | | |  |  |  |
|  |  |  | | *c* | | 0.77 | <0.001 |  | | |  |  |  |
|  |  |  | | *c’* | | 0.85 | <0.001 |  | | |  |  |  |

An intention-to-treat (ITT) approach was used, using the Full Information Maximum Likelihood (FIML) to address missing data. The independent variable is the group condition (MBCT-TiF vs OMP). The potential mediator (mindfulness, decentering, or self-compassion) was based on T1-T2 [week 4 to week 8] residualised change scores. The dependent variable (outcome) is psychological quality of life at T3 [week 12]. Models controlled for the outcome at baseline [T0]. Path coefficients are (standardized) ordinary least squares-based regression coefficients. *a*: direct path between the independent variable and the mediator. *b*: direct path between the mediator and the outcome. The product of “*ab*” is the bootstrapped indirect effect (IE) using 10,000 samples. *c*: direct effect of the independent variable on the dependent variable after adjustment for the mediating effects. *c*’: total effects. *R*^2^: variance explained by regression models. *F*: Snedecor’s *F* associated with the adjustment of the regression model. *Coeff*: (standardized) slope. *t*: Student’s *t* associated with the slope using the Wald test. *SE*: standard error. *p*^a^: *p*-value related to *F*-test. *p*^b^: *p*-value related to *t*-test. 95% CI: 95% confidence interval.

**Supplement E**. Direct and bootstrapped indirect effects in the simple mediation path analysis models of depression

|  |  |  |  | | **DIRECT EFFECTS** | | | |  | **INDIRECT EFFECTS** | | | |
| --- | --- | --- | --- | --- | --- | --- | --- | --- | --- | --- | --- | --- | --- |
| **Mediators** | ***R^2^*** | ***p^a^*** | | ***path*** | | ***Coeff.*** | ***p^b^*** |  | | | ***path*** | ***Coeff.*** | ***95%CI*** |
| Mindfulness | 0.14 | 0.027 | | *a* | | 0.50 | 0.001 |  | | | *ab* | -0.11 | -0.26, -0.03 |
|  |  |  | | *b* | | -0.23 | 0.009 |  | | |  |  |  |
|  |  |  | | *c* | | -0.50 | 0.001 |  | | |  |  |  |
|  |  |  | | *c’* | | -0.61 | <0.001 |  | | |  |  |  |
| Decentering | 0.12 | 0.050 | | *a* | | 0.60 | <0.001 |  | | | *ab* | -0.11 | -0.27, -0.01 |
|  |  |  | | *b* | | -0.18 | 0.046 |  | | |  |  |  |
|  |  |  | | *c* | | -0.49 | 0.001 |  | | |  |  |  |
|  |  |  | | *c’* | | -0.60 | <0.001 |  | | |  |  |  |
| Self-compassion | 0.14 | 0.029 | | *a* | | 0.45 | 0.005 |  | | | *ab* | -0.10 | -0.25, -0.02 |
|  |  |  | | *b* | | -0.22 | 0.012 |  | | |  |  |  |
|  |  |  | | *c* | | -0.51 | 0.001 |  | | |  |  |  |
|  |  |  | | *c’* | | -0.61 | <0.001 |  | | |  |  |  |

An intention-to-treat (ITT) approach was used, using the Full Information Maximum Likelihood (FIML) to address missing data. The independent variable is the group condition (MBCT-TiF vs OMP). The potential mediator (mindfulness, decentering, or self-compassion) was based on T1-T2 [week 4 to week 8] residualised change scores. The dependent variable (outcome) is depression of life at T3 [week 12]. Models controlled for the outcome at baseline [T0]. Path coefficients are (standardized) ordinary least squares-based regression coefficients. *a*: direct path between the independent variable and the mediator. *b*: direct path between the mediator and the outcome. The product of “*ab*” is the bootstrapped indirect effect (IE) using 10,000 samples. *c*: direct effect of the independent variable on the dependent variable after adjustment for the mediating effects. *c*’: total effects. *R*^2^: variance explained by regression models. *F*: Snedecor’s *F* associated with the adjustment of the regression model. *Coeff*: (standardized) slope. *t*: Student’s *t* associated with the slope using the Wald test. *SE*: standard error. *p*^a^: *p*-value related to *F*-test. *p*^b^: *p*-value related to *t*-test. 95% CI: 95% confidence interval.

**Supplement F**. Direct and bootstrapped indirect effects in the simple mediation path analysis models of anxiety

|  |  |  |  | | **DIRECT EFFECTS** | | | |  | **INDIRECT EFFECTS** | | | |
| --- | --- | --- | --- | --- | --- | --- | --- | --- | --- | --- | --- | --- | --- |
| **Mediators** | ***R^2^*** | ***p^a^*** | | ***path*** | | ***Coeff.*** | ***p^b^*** |  | | | ***path*** | ***Coeff.*** | ***95%CI*** |
| Mindfulness | 0.13 | 0.023 | | *a* | | 0.49 | 0.002 |  | | | *ab* | -0.09 | -0.23, -0.01 |
|  |  |  | | *b* | | -0.18 | 0.026 |  | | |  |  |  |
|  |  |  | | *c* | | -0.55 | <0.001 |  | | |  |  |  |
|  |  |  | | *c’* | | -0.64 | <0.001 |  | | |  |  |  |
| Decentering | 0.15 | 0.020 | | *a* | | 0.60 | <0.001 |  | | | *ab* | -0.14 | -0.32, -0.03 |
|  |  |  | | *b* | | -0.23 | 0.012 |  | | |  |  |  |
|  |  |  | | *c* | | -0.49 | 0.001 |  | | |  |  |  |
|  |  |  | | *c’* | | -0.63 | <0.001 |  | | |  |  |  |
| Self-compassion | 0.12 | 0.031 | | *a* | | 0.44 | 0.006 |  | | | *ab* | -0.05 | -0.17, 0.01 |
|  |  |  | | *b* | | -0.12 | 0.139 |  | | |  |  |  |
|  |  |  | | *c* | | -0.58 | <0.001 |  | | |  |  |  |
|  |  |  | | *c’* | | -0.64 | <0.001 |  | | |  |  |  |

An intention-to-treat (ITT) approach was used, using the Full Information Maximum Likelihood (FIML) to address missing data. The independent variable is the group condition (MBCT-TiF vs OMP). The potential mediator (mindfulness, decentering, or self-compassion) was based on T1-T2 [week 4 to week 8] residualised change scores. The dependent variable (outcome) is anxiety of life at T3 [week 12]. Models controlled for the outcome at baseline [T0]. Path coefficients are (standardized) ordinary least squares-based regression coefficients. *a*: direct path between the independent variable and the mediator. *b*: direct path between the mediator and the outcome. The product of “*ab*” is the bootstrapped indirect effect (IE) using 10,000 samples. *c*: direct effect of the independent variable on the dependent variable after adjustment for the mediating effects. *c*’: total effects. *R*^2^: variance explained by regression models. *F*: Snedecor’s *F* associated with the adjustment of the regression model. *Coeff*: (standardized) slope. *t*: Student’s *t* associated with the slope using the Wald test. *SE*: standard error. *p*^a^: *p*-value related to *F*-test. *p*^b^: *p*-value related to *t*-test. 95% CI: 95% confidence interval.

**Supplement G.** Direct and bootstrapped indirect effects in the simple (reversed) mediation path analysis models on mindfulness

|  |  |  |  | | **DIRECT EFFECTS** | | | |  | **INDIRECT EFFECTS** | | | |
| --- | --- | --- | --- | --- | --- | --- | --- | --- | --- | --- | --- | --- | --- |
| **Mediators** | ***R^2^*** | ***p^a^*** | | ***path*** | | ***Coeff.*** | ***p^b^*** |  | | | ***path*** | ***Coeff.*** | ***95%CI*** |
| Mental Well-being | 0.35 | <0.001 | | *a* | | 0.32 | 0.049 |  | | | *ab* | 0.08 | 0.003, 0.20 |
|  |  |  | | *b* | | 0.24 | 0.001 |  | | |  |  |  |
|  |  |  | | *c* | | 0.99 | <0.001 |  | | |  |  |  |
|  |  |  | | *c’* | | 1.07 | <0.001 |  | | |  |  |  |
| Psychological Quality of Life | 0.37 | <0.001 | | *a* | | 0.50 | 0.002 |  | | | *ab* | 0.14 | 0.04, 0.30 |
|  |  |  | | *b* | | 0.29 | <0.001 |  | | |  |  |  |
|  |  |  | | *c* | | 0.93 | <0.001 |  | | |  |  |  |
|  |  |  | | *c’* | | 1.08 | <0.001 |  | | |  |  |  |
| Depression | 0.42 | <0.001 | | *a* | | -0.20 | 0.216 |  | | | *ab* | 0.07 | -0.04, 0.21 |
|  |  |  | | *b* | | -0.36 | <0.001 |  | | |  |  |  |
|  |  |  | | *c* | | 0.99 | <0.001 |  | | |  |  |  |
|  |  |  | | *c’* | | 1.06 | <0.001 |  | | |  |  |  |
| Anxiety | 0.37 | <0.001 | | *a* | | -0.30 | 0.064 |  | | | *ab* | 0.09 | 0.001, 0.21 |
|  |  |  | | *b* | | -0.29 | <0.001 |  | | |  |  |  |
|  |  |  | | *c* | | 0.99 | <0.001 |  | | |  |  |  |
|  |  |  | | *c’* | | 1.08 | <0.001 |  | | |  |  |  |

An intention-to-treat (ITT) approach was used, using the Full Information Maximum Likelihood (FIML) to address missing data. The independent variable is the group condition (MBCT-TiF vs OMP). The potential mediator (mental well-being, psychological quality of life, depression, and anxiety) was based on T1-T2 [week 4 to week 8] residualised change scores. The dependent variable (outcome) is mindfulness at T3 [week 12]. Models controlled for the outcome at baseline [T0]. Path coefficients are (standardized) ordinary least squares-based regression coefficients. *a*: direct path between the independent variable and the mediator. *b*: direct path between the mediator and the outcome. The product of “*ab*” is the bootstrapped indirect effect (IE) using 10,000 samples. *c*: direct effect of the independent variable on the dependent variable after adjustment for the mediating effects. *c*’: total effects. *R*^2^: variance explained by regression models. *F*: Snedecor’s *F* associated with the adjustment of the regression model. *Coeff*: (standardized) slope. *t*: Student’s *t* associated with the slope using the Wald test. *SE*: standard error. *p*^a^: *p*-value related to *F*-test. *p*^b^: *p*-value related to *t*-test. 95% CI: 95% confidence interval.

**Supplement H.** Direct and bootstrapped indirect effects in the simple (reversed) mediation path analysis models on decentering

|  |  |  |  | | **DIRECT EFFECTS** | | | |  | **INDIRECT EFFECTS** | | | |
| --- | --- | --- | --- | --- | --- | --- | --- | --- | --- | --- | --- | --- | --- |
| **Mediators** | ***R^2^*** | ***p^a^*** | | ***path*** | | ***Coeff.*** | ***p^b^*** |  | | | ***path*** | ***Coeff.*** | ***95%CI*** |
| Mental Well-being | 0.38 | <0.001 | | *a* | | 0.32 | 0.052 |  | | | *ab* | 0.09 | 0.01, 0.21 |
|  |  |  | | *b* | | 0.29 | <0.001 |  | | |  |  |  |
|  |  |  | | *c* | | 0.98 | <0.001 |  | | |  |  |  |
|  |  |  | | *c’* | | 1.07 | <0.001 |  | | |  |  |  |
| Psychological Quality of Life | 0.38 | <0.001 | | *a* | | 0.50 | 0.002 |  | | | *ab* | 0.15 | 0.06, 0.29 |
|  |  |  | | *b* | | 0.30 | <0.001 |  | | |  |  |  |
|  |  |  | | *c* | | 0.93 | <0.001 |  | | |  |  |  |
|  |  |  | | *c’* | | 1.08 | <0.001 |  | | |  |  |  |
| Depression | 0.34 | <0.001 | | *a* | | -0.20 | 0.230 |  | | | *ab* | 0.04 | -0.02, 0.15 |
|  |  |  | | *b* | | -0.21 | 0.006 |  | | |  |  |  |
|  |  |  | | *c* | | 1.03 | <0.001 |  | | |  |  |  |
|  |  |  | | *c’* | | 1.07 | <0.001 |  | | |  |  |  |
| Anxiety | 0.34 | <0.001 | | *a* | | -0.30 | 0.068 |  | | | *ab* | 0.07 | 0.001, 0.18 |
|  |  |  | | *b* | | -0.22 | 0.001 |  | | |  |  |  |
|  |  |  | | *c* | | 1.02 | <0.001 |  | | |  |  |  |
|  |  |  | | *c’* | | 1.08 | <0.001 |  | | |  |  |  |

An intention-to-treat (ITT) approach was used, using the Full Information Maximum Likelihood (FIML) to address missing data. The independent variable is the group condition (MBCT-TiF vs OMP). The potential mediator (mental well-being, psychological quality of life, depression, and anxiety) was based on T1-T2 [week 4 to week 8] residualised change scores. The dependent variable (outcome) is decentering at T3 [week 12]. Models controlled for the outcome at baseline [T0]. Path coefficients are (standardized) ordinary least squares-based regression coefficients. *a*: direct path between the independent variable and the mediator. *b*: direct path between the mediator and the outcome. The product of “*ab*” is the bootstrapped indirect effect (IE) using 10,000 samples. *c*: direct effect of the independent variable on the dependent variable after adjustment for the mediating effects. *c*’: total effects. *R*^2^: variance explained by regression models. *F*: Snedecor’s *F* associated with the adjustment of the regression model. *Coeff*: (standardized) slope. *t*: Student’s *t* associated with the slope using the Wald test. *SE*: standard error. *p*^a^: *p*-value related to *F*-test. *p*^b^: *p*-value related to *t*-test. 95% CI: 95% confidence interval.

**Supplement I.** Direct and bootstrapped indirect effects in the simple (reversed) mediation path analysis models on self-compassion

|  |  |  |  | | **DIRECT EFFECTS** | | | |  | **INDIRECT EFFECTS** | | | |
| --- | --- | --- | --- | --- | --- | --- | --- | --- | --- | --- | --- | --- | --- |
| **Mediators** | ***R^2^*** | ***p^a^*** | | ***path*** | | ***Coeff.*** | ***p^b^*** |  | | | ***path*** | ***Coeff.*** | ***95%CI*** |
| Mental Well-being | 0.34 | <0.001 | | *a* | | 0.32 | 0.047 |  | | | *ab* | 0.08 | 0.01, 0.18 |
|  |  |  | | *b* | | 0.24 | <0.001 |  | | |  |  |  |
|  |  |  | | *c* | | 0.99 | <0.001 |  | | |  |  |  |
|  |  |  | | *c’* | | 1.06 | <0.001 |  | | |  |  |  |
| Psychological Quality of Life | 0.35 | <0.001 | | *a* | | 0.50 | 0.002 |  | | | *ab* | 0.12 | 0.05, 0.24 |
|  |  |  | | *b* | | 0.25 | <0.001 |  | | |  |  |  |
|  |  |  | | *c* | | 0.95 | <0.001 |  | | |  |  |  |
|  |  |  | | *c’* | | 1.07 | <0.001 |  | | |  |  |  |
| Depression | 0.37 | <0.001 | | *a* | | -0.21 | 0.214 |  | | | *ab* | 0.06 | -0.03, 0.16 |
|  |  |  | | *b* | | -0.28 | <0.001 |  | | |  |  |  |
|  |  |  | | *c* | | 1.00 | <0.001 |  | | |  |  |  |
|  |  |  | | *c’* | | 1.06 | <0.001 |  | | |  |  |  |
| Anxiety | 0.36 | <0.001 | | *a* | | -0.30 | 0.063 |  | | | *ab* | 0.08 | 0.001, 0.19 |
|  |  |  | | *b* | | -0.26 | <0.001 |  | | |  |  |  |
|  |  |  | | *c* | | 0.99 | <0.001 |  | | |  |  |  |
|  |  |  | | *c’* | | 1.07 | <0.001 |  | | |  |  |  |

An intention-to-treat (ITT) approach was used, using the Full Information Maximum Likelihood (FIML) to address missing data. The independent variable is the group condition (MBCT-TiF vs OMP). The potential mediator (mental well-being, psychological quality of life, depression, and anxiety) was based on T1-T2 [week 4 to week 8] residualised change scores. The dependent variable (outcome) is self-compassion at T3 [week 12]. Models controlled for the outcome at baseline [T0]. Path coefficients are (standardized) ordinary least squares-based regression coefficients. *a*: direct path between the independent variable and the mediator. *b*: direct path between the mediator and the outcome. The product of “*ab*” is the bootstrapped indirect effect (IE) using 10,000 samples. *c*: direct effect of the independent variable on the dependent variable after adjustment for the mediating effects. *c*’: total effects. *R*^2^: variance explained by regression models. *F*: Snedecor’s *F* associated with the adjustment of the regression model. *Coeff*: (standardized) slope. *t*: Student’s *t* associated with the slope using the Wald test. *SE*: standard error. *p*^a^: *p*-value related to *F*-test. *p*^b^: *p*-value related to *t*-test. 95% CI: 95% confidence interval.
